# The severity of meiotic aneuploidy is associated with altered morphokinetic variables of mouse oocyte maturation

**DOI:** 10.1093/hropen/hoae023

**Published:** 2024-04-23

**Authors:** Yiru Zhu, Catherine R Kratka, Jeffrey Pea, Hoi Chang Lee, Caroline E Kratka, Jia Xu, Diego Marin, Nathan R Treff, Francesca E Duncan

**Affiliations:** Department of Obstetrics and Gynecology, Feinberg School of Medicine, Northwestern University, Chicago, IL, USA; Genomic Prediction Inc., North Brunswick Township, NJ, USA; Department of Obstetrics and Gynecology, Feinberg School of Medicine, Northwestern University, Chicago, IL, USA; Department of Obstetrics and Gynecology, Feinberg School of Medicine, Northwestern University, Chicago, IL, USA; Department of Obstetrics and Gynecology, Feinberg School of Medicine, Northwestern University, Chicago, IL, USA; Genomic Prediction Inc., North Brunswick Township, NJ, USA; Genomic Prediction Inc., North Brunswick Township, NJ, USA; Department of Genetics, Rutgers University, New Brunswick, NJ, USA; Genomic Prediction Inc., North Brunswick Township, NJ, USA; Department of Obstetrics, Gynecology, and Reproductive Sciences, Rutgers University, New Brunswick, NJ, USA; Department of Obstetrics and Gynecology, Feinberg School of Medicine, Northwestern University, Chicago, IL, USA

**Keywords:** oocyte quality, aneuploidy, meiosis, chromosomal abnormalities, time-lapse microscopy, *in vitro* maturation

## Abstract

**STUDY QUESTION:**

Is there an association between morphokinetic variables of meiotic maturation and the severity of aneuploidy following *in vitro* maturation (IVM) in the mouse?

**SUMMARY ANSWER:**

The severity of meiotic aneuploidy correlates with an extended time to first polar body extrusion (tPB1) and duration of meiosis I (dMI).

**WHAT IS KNOWN ALREADY:**

Morphokinetic variables measured using time-lapse technology allow for the non-invasive evaluation of preimplantation embryo development within clinical assisted reproductive technology (ART). We recently applied this technology to monitor meiotic progression during IVM of mouse gametes. Whether there is a relationship between morphokinetic variables of meiotic progression and aneuploidy in the resulting egg has not been systematically examined at the resolution of specific chromosomes. Next-generation sequencing (NGS) is a robust clinical tool for determining aneuploidy status and has been reverse-translated in mouse blastocysts and oocytes. Therefore, we harnessed the technologies of time-lapse imaging and NGS to determine the relationship between the morphokinetics of meiotic progression and egg aneuploidy.

**STUDY DESIGN, SIZE, DURATION:**

Cumulus–oocyte complexes were collected from large antral follicles from hyperstimulated CD-1 mice. Cumulus cells were removed, and spontaneous IVM was performed in the absence or presence of two doses of Nocodazole (25 or 50 nM) to induce a spectrum of spindle abnormalities and chromosome segregation errors during oocyte meiosis. Comprehensive chromosome screening was then performed in the resulting eggs, and morphokinetic variables and ploidy status were compared across experimental groups (control, n = 11; 25 nM Nocodazole, n = 13; 50 nM Nocodazole, n = 23).

**PARTICIPANTS/MATERIALS, SETTING, METHODS:**

We monitored IVM in mouse oocytes using time-lapse microscopy for 16 h, and time to germinal vesicle breakdown (tGVBD), tPB1, and dMI were analyzed. Following IVM, comprehensive chromosome screening was performed on the eggs and their matched first polar bodies via adaptation of an NGS-based preimplantation genetic testing for aneuploidy (PGT-A) assay. Bioinformatics analysis was performed to align reads to the mouse genome and determine copy number-based predictions of aneuploidy. The concordance of each polar body–egg pair (reciprocal errors) was used to validate the results. Ploidy status was categorized as euploid, 1–3 chromosomal segregation errors, or ≥4 chromosomal segregation errors. Additionally, aneuploidy due to premature separation of sister chromatids (PSSC) versus non-disjunction (NDJ) was distinguished.

**MAIN RESULTS AND THE ROLE OF CHANCE:**

We applied and validated state-of-the-art NGS technology to screen aneuploidy in individual mouse eggs and matched polar bodies at the chromosome-specific level. By performing IVM in the presence of different doses of Nocodazole, we induced a range of aneuploidy. No aneuploidy was observed in the absence of Nocodazole (0/11), whereas IVM in the presence of 25 and 50 nM Nocodazole resulted in an aneuploidy incidence of 7.69% (1/13) and 82.61% (19/23), respectively. Of the aneuploid eggs, 5% (1/20) was due to PSSC, 65% (13/20) to NDJ, and the remainder to a combination of both. There was no relationship between ploidy status and tGVBD, but tPB1 and the dMI were both significantly prolonged in eggs with reciprocal aneuploidy events compared to the euploid eggs, and this scaled with the severity of aneuploidy. Eggs with ≥4 aneuploid chromosomes had the longest tPB1 and dMI (*P* < 0.0001), whereas eggs with one to three aneuploid chromosomes exhibited intermediate lengths of time (*P* < 0.0001).

**LARGE SCALE DATA:**

N/A.

**LIMITATIONS, REASONS FOR CAUTION:**

We used Nocodazole in this study to disrupt the meiotic spindle and induce aneuploidy in mouse oocytes. Whether the association between morphokinetic variables of meiotic progression and the severity of aneuploidy occurs with other compounds that induce chromosome segregation errors remain to be investigated. In addition, unlike mouse oocytes, human IVM requires the presence of cumulus cells, which precludes visualization of morphokinetic variables of meiotic progression. Thus, our study may have limited direct clinical translatability.

**WIDER IMPLICATIONS OF THE FINDINGS:**

We validated NGS in mouse eggs to detect aneuploidy at a chromosome-specific resolution which greatly improves the utility of the mouse model. With a tractable and validated model system for characterizing meiotic aneuploidy, investigations into the molecular mechanisms and factors which may influence aneuploidy can be further elaborated. Time-lapse analyses of morphokinetic variables of meiotic progression may be a useful non-invasive predictor of aneuploidy severity.

**STUDY FUNDING/COMPETING INTERESTS:**

This work was supported by the Bill & Melinda Gates Foundation (INV-003385). Under the grant conditions of the Foundation, a Creative Commons Attribution 4.0 Generic License has already been assigned to the Author Accepted Manuscript version that might arise from this submission. The authors have no conflict of interest to disclose.

WHAT DOES THIS MEAN FOR PATIENTS? Closed time-lapse imaging systems are commonly used in assisted reproductive technology settings to monitor embryo development with minimal disruption, and to measure the timing of key developmental events based on morphology to generate ‘morphokinetic’ markers. These morphokinetic markers have been used to non-invasively predict reproductive outcomes such as implantation and pregnancy. However, this approach has been largely limited to embryos and assessment of their quality. The purpose of this study is to expand the utility of morphokinetic markers to comprehensively evaluate oocyte quality. In this study, mouse oocytes were *in vitro* matured in a time-lapse incubator to evaluate progression of meiosis. Meiosis refers to a specialized series of cell divisions which are necessary to reduce the number of chromosomes in the female gamete to a single set (haploid) in preparation for fertilization with sperm. Oocytes were also matured in the presence of Nocodazole, a drug known to inhibit accurate organization and separation of chromosomes and, thereby, cause the presence or absence of extra chromosomes (aneuploidy). Morphokinetic variables of meiotic progression were analyzed, and mature eggs were then screened for aneuploidy using next-generation sequencing. We found that morphokinetic variables with a prolonged time to complete meiosis I were associated with an increased severity of aneuploidy. Thus, time-lapse variables of meiotic progression may be a useful non-invasive predictor of oocyte quality that has both clinical and pharmaceutical applications.

## Introduction

In clinical ART settings, closed time-lapse monitoring systems have enabled the observation and evaluation of preimplantation embryo development within a stable incubation environment with respect to temperature, pH, and oxygen levels. Time-lapse imaging technology integrates an inverted microscope and digital camera with a closed incubator system to ensure that observations can be made without disrupting embryo development (Cruz *et al.*, 2011). In addition to facilitating morphological grading of embryos, these systems generate continuous time-lapse images which allow embryologists to accurately evaluate the timing of key events in embryo development. The establishment of morphokinetic variables and their integration into computational algorithms have been used to predict ART outcomes to various degrees of success, including blastocyst formation, implantation, and pregnancy (Lemmen *et al.*, 2008; Wong *et al.*, 2010; Meseguer *et al.*, 2012; Desai *et al.*, 2014; Rubio *et al.*, 2014). Morphokinetic analysis using closed time-lapse monitoring systems to date has been largely limited to preimplantation embryos in the clinical ART setting.

Recently, we extended the use of this technology to monitor oocyte meiotic maturation using the mouse model (Suebthawinkul *et al.*, 2022, 2023). During ovulation, oocytes resume meiosis in response to the luteinizing hormone surge and progress from the dictyate arrest in prophase of meiosis I (MI) to the metaphase stage of meiosis II (MII) (Sánchez and Smitz, 2012). This transition is characterized by key events that can be clearly visualized morphologically by transmitted light microscopy, including the breakdown of the nuclear envelope or germinal vesicle breakdown (GVBD) and extrusion of the first polar body (PBI) (Sánchez and Smitz, 2012; Gilchrist *et al.*, 2016; Pan and Li, 2019; Turathum *et al.*, 2021). We have established baseline morphokinetic variables of meiotic progression and demonstrated that they were sensitive to perturbation (Suebthawinkul *et al.*, 2022). Thus, this time-lapse monitoring system has the potential to reveal correlations between morphokinetic variables of IVM and clinically relevant outcomes, such as egg aneuploidy or embryo development. Such morphokinetic variables may, therefore, ultimately have utility as a non-invasive marker of oocyte quality.

The goal of this study was to evaluate the relationship between morphokinetic variables of oocyte meiotic progression and aneuploidy status. To accomplish this, we used Nocodazole, a known microtubule-disrupting compound, to induce aneuploidy in oocytes that were *in vitro* matured in the EmbryoScope+™ system. We then performed whole-genome amplification (WGA) and next-generation sequencing (NGS) on the resulting MII eggs derived from either the control or Nocodazole groups to obtain the ploidy status at chromosome-specific resolution. We also determined whether there was an association between the morphokinetic variables of meiotic progression and the severity of aneuploidy. We found that Nocodazole induced aneuploidy due to both non-disjunction (NDJ) and premature separation of sister chromatids (PSSC), and the degree of aneuploidy was dose-dependent. Furthermore, although the timing of GVBD was not affected by aneuploidy, the timing of polar body extrusion and, thereby, the duration of MI was significantly longer in aneuploid versus euploid eggs. The relationship between morphokinetics of meiotic progression and aneuploidy has potential implications for the study of meiotic mechanisms, drug screening for novel female contraceptives, and clinical evaluations in ART settings.

## Materials and methods

### Animals

Reproductively young adult CD-1 female mice were obtained from Envigo (Indianapolis, IN, USA). Upon arrival, mice were allowed to acclimatize for at least a week before being used for downstream experiments between 6 and 12 weeks of age. Mice were housed in a controlled barrier facility at the Northwestern University Center for Comparative Medicine under constant temperature, humidity, and light (14 h light/10 h dark). Mice were provided food and water *ad libitum*. Mice were fed Teklad Global 2916 chow (Envigo), a diet that excludes soybean and alfalfa meal to minimize exposure to phytoestrogens. Mice were used for oocyte collection as described below. All animal experiments described were approved by the Institutional Animal Care and Use Committee and performed under the National Institutes of Health Guidelines.

### Ovarian stimulation and oocyte collection

Eight mice were stimulated with intraperitoneal injections of 5 IU pregnant mare serum gonadotropin (PMSG) (ProSpec-Tany TechnoGene, East Brunswick, NJ, USA, Cat No. HOR-272), and ovaries were harvested 44–46 h post-PMSG-injection. After dissection, isolated ovaries were transferred into dishes containing pre-warmed Leibovitz’s medium (L15) (Life Technologies Corporation, Grand Island, NY, USA) supplemented with 3 mg/ml polyvinylpyrrolidone (PVP) (Sigma-Aldrich, St Louis, MO, USA) and 0.5% (v/v) Penicillin–Streptomycin (PS) (Life Technologies Corporation) (L15/PVP/PS). Cumulus–oocyte complexes (COCs) were collected following mechanical disruption of antral follicles with insulin syringes and transferred to L15/PVP/PS media supplemented with 2.5 μM milrinone (Sigma-Aldrich), a phosphodiesterase 3A (PDE3A) inhibitor, to maintain oocyte meiotic arrest. Cumulus cells surrounding the COCs were removed by mechanical disruption to obtain denuded oocytes. After denuding, the isolated oocytes were transferred to pre-equilibrated α-MEM+GlutaMAX (Thermo Fisher Scientific, Waltham, MA, USA)/PS/bovine serum albumin (BSA) (Sigma-Aldrich) (α-MEM/PS/BSA) media supplemented with 2.5 μM milrinone, to maintain meiotic arrest, and allowed to recover from the collection process for 1 h at 37 °C in a humidified atmosphere of 5% CO_2_ in air before being loaded into the EmbryoScope+™ specific culture dish, EmbryoSlide (Vitrolife, Denver, CO, USA). Denuded oocytes were pooled together from four mice per experiment to minimize animal-specific variability.

### IVM within EmbryoSlide and EmbryoScope+™ ± Nocodazole treatment

**Figure 1.**
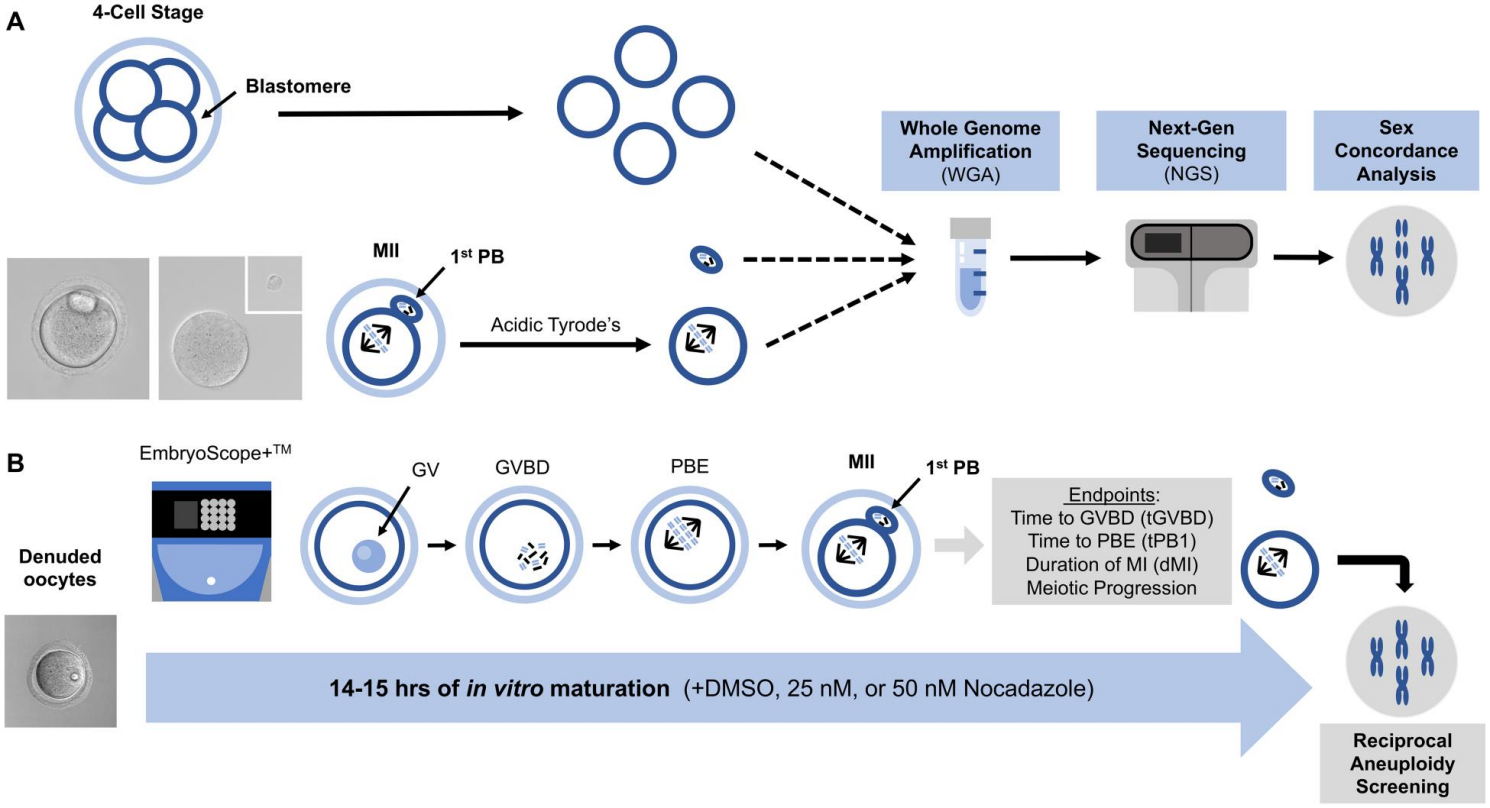
**Overview of using whole genome amplification (WGA) and next generation sequencing (NGS) for conducting sex concordance and reciprocal aneuploidy analysis**. (**A**) Commercially available mouse blastomeres and matched meiosis II (MII) oocyte/polar body pairs were used for platform validation. The oocyte and its matched polar body were separated following removal of the zona pellucida following treatment with Acidic Tyrode’s solution. The number of X and Y chromosomes were compared among different blastomeres of the same mouse embryo or with matched oocyte/polar body pairs following WGA and NGS. (**B**) IVM was performed on immature oocytes at GV stage in the EmbryoScope+™ with either DMSO (control), 25 or 50 nM of Nocodazole. After IVM, morphokinetic variables were determined and reciprocal aneuploidy analysis was conducted on MII oocytes to determine the changes in chromatid copy number. WGA, whole-genome amplification; NGS, next-generation sequencing; GV, germinal vesicle; GVBD, germinal vesicle breakdown; PBE, polar body extrusion; MI, meiosis I.

EmbryoSlide microwells were loaded with α-MEM/BSA/PS media and overlayed with 1.6 ml of mineral oil (Sigma-Aldrich) the day before the experiment to allow the dishes to equilibrate in the EmbryoScope+™ for 9–11 h. Prior to loading into the EmbryoSlides, denuded oocytes were washed together in a new dish containing L15/PVP/PS without milrinone. Removal of milrinone allows for degradation of cyclic adenosine monophosphate (cAMP), which initiates synchronous and spontaneous meiotic resumption. Denuded oocytes were then loaded into microwells of the EmbryoSlide and IVM was performed in α-MEM/PS/BSA media for a total of 14–15 h at 37 °C in a humidified atmosphere of 5% CO_2_ in air (Fig. 1B). The EmbryoScope+™ can accommodate simultaneous and continuous monitoring of 240 samples in parallel and eliminates the need to image samples outside of the incubator. Images were taken every 10–20 min across 11 focal planes separated by 10 µm in the *z*-axis with a 12-bit monochrome CMOS camera and a single red LED. This technology is used in clinical ART and is safe for human gametes and preimplantation embryos (Meseguer *et al.*, 2012; ESHRE Working Group on Time-Lapse Technology *et al.*, 2020). Moreover, we have optimized the use of EmbryoScope+™ for mouse oocyte IVM (Suebthawinkul *et al.*, 2022, 2023). Two operators washed and loaded the oocytes at the same time with an estimated 5–10 min interval between each dish.

To induce aneuploidy, we performed IVM in the presence of Nocodazole. This drug depolymerizes microtubules, disrupts the meiotic spindle, and results in chromosome misalignment in a dose-dependent fashion (Wassmann *et al.*, 2003; Sun *et al.*, 2005; Suebthawinkul *et al.*, 2022). IVM was performed as described above in the presence of 25 or 50 nM of Nocodazole prepared from a 0.5 mM stock solution in dimethyl sulfoxide (DMSO) (Sigma-Aldrich, Cat No. M1404) (Fig. 1B). The same volume of DMSO as in the 50 nM group was added to the media as the control group. This experiment was performed twice with a total of between 11–23 oocytes per treatment group. The gametes were then evaluated for maturation status, morphokinetic variables of meiotic progression, and reciprocal aneuploidy screening in the egg and matched polar body, as described below (Fig. 1B). We removed eggs from further analysis based on exclusion criteria, including presence of cytoplasmic vacuoles within the eggs, loss of PB when processing samples for NGS, or validation of statistical outliers using the Grubbs’ test. From the remaining pool, we ultimately submitted a total of 11 control eggs for NGS due to cost limitations.

### Analysis of oocyte maturation status and morphokinetic variables of meiotic progression

Following IVM, the meiotic maturation status of denuded oocytes in each treatment group was assessed based on morphological criteria following review of the time-lapse imaging data using the EmbryoViewer software (Vitrolife). Oocytes that failed to mature and remained arrested at prophase of MI were characterized by an intact nucleus or germinal vesicle (GV oocyte), whereas mature eggs arrested at metaphase of MII (MII egg) were characterized by extrusion of the PB1. Gametes that lacked both a GV or PB had undergone GVBD and were at either pro-metaphase I or metaphase I. The percentage of oocytes at each stage of meiosis was reported in all experiments.

Morphokinetic variables of meiotic progression, including time to GVBD (tGVBD), time to first polar body extrusion (tPB1), and duration of MI (dMI), were determined as previously described (Suebthawinkul *et al.*, 2022). In brief, t0 was set as the time when denuded oocytes were placed into the EmbryoScope+™ for IVM and imaging was started. tGVBD was defined as the time point when the complete loss of the GV was first observed (Supplementary Fig. S1), and tPB1 was defined as the time point when cytokinesis was complete and there was clear separation between the PB and oocyte plasma membranes (Supplementary Fig. S2). dMI was defined as the time difference between tGVBD and tPB1 (tPB1–tGVBD). All annotations were conducted by a single rater and double-checked by another experienced rater based on our defined scoring criteria. Raters were not blinded to the treatment groups during the annotation process. Morphokinetic variables were calculated and plotted individually for each oocyte. Time points for morphokinetic variables of the control group were generally similar to those previously published by our group (Suebthawinkul *et al.*, 2022, 2023). In addition, within each study, the same raters scored all samples across the entire study.

### Immunofluorescence for cytoskeleton analysis

To assess the cytoskeleton following IVM, oocytes that reached MII stage were fixed in 3.8% paraformaldehyde (Electron Microscopy Sciences, Hatfield, PA, USA) with 0.1% Triton X-100 (TX-100) (Sigma-Aldrich) for 25 min at 37 °C followed by washing with blocking buffer (1× PBS, 0.01% Tween-20 (Sigma-Aldrich), 0.02% sodium azide (NaN_3_) (Sigma-Aldrich), and 0.3% BSA) four times for 5 min. The gametes were transferred to permeabilization solution (1× PBS, 0.1% TX-100, 0.02% NaN_3_, and 0.3% BSA) for 15 min at room temperature and washed again with blocking buffer twice for 5 min each wash before staining. To visualize actin and microtubules, the gametes were incubated with Rhodamine Phalloidin (1:50; Invitrogen, Waltham, MA, USA, Cat No. R415) and α-tubulin (11H10) Rabbit mAB 488 (1:100; Cell Signaling Technology, Danvers, MA, USA, Cat No. 5063S) for 2 h at room temperature. Gametes were then rinsed with blocking buffer three times for 20 min each wash and mounted on slides in Vectashield Antifade Mounting Medium with DAPI (4, 6-diamidino-2-phenylindole; Vector Laboratories, Burlingame, CA, USA). Gametes were imaged at 40× magnification on a Leica SP5 inverted laser scanning confocal microscope (Leica Microsystems, Wetzlar, Germany) using 405, 488, and 543 nm lasers. All images were processed using LAS AF (Leica Microsystems) and analyzed using FIJI (National Institutes of Health, Bethesda, MD, USA).

### Whole-genome amplification and NGS

To obtain matched egg and polar body samples for analysis, intact mouse oocytes that reached the metaphase of MII after IVM were briefly incubated in acidified Tyrode’s solution (EMD Millipore, Billerica, MA, USA) to remove the zona pellucida. The PB was gently aspirated from the egg, and matched polar body and egg samples were washed in hypotonic wash buffer (HWB) and snap-frozen individually in a sterile PCR tube containing 1 μl of HWB on a dry ice and ethanol bath. Mouse preimplantation embryos at the 4-cell stage were purchased from Embryotech Laboratories (Haverhill, MA, USA) to obtain male and female blastomeres for validation of the comprehensive chromosome screening technology. Three or four blastomeres per mouse embryo (n* *=* *10) were collected, washed, and loaded into PCR tubes as described below.

The DNA from mouse blastomeres and de-identified egg/polar body pairs was extracted and amplified using the Ion SingleSeq Kit (ThermoFisher Scientific). Amplified samples were run on E-Gel™ 48 agarose gels with the E-Gel™ Low Range Quantitative DNA ladder (ThermoFisher Scientific) to visualize the expected 350 base pair fragment sizes. Pooled amplified libraries were purified using AMPure™ XP beads (Beckman Coulter, Pasadena, CA, USA) and quantified using the Qubit™ dsDNA HS (High Sensitivity) Assay Kit with the Qubit 3.0 Fluorometer (ThermoFisher Scientific). Human genomic DNA (hgDNA) obtained from the Genomic Prediction Clinical Library (North Brunswick, NJ, USA) was used as a positive control. WGA DNA was normalized to 1 nM using a Low TE buffer (Fisher Scientific, Pittsburgh, PA, USA) and further diluted to 80 pM using nuclease-free water before being transferred into the Ion S5™ ExT Chef Reagents cartridge (ThermoFisher Scientific). The pooled libraries underwent emulsion PCR and enrichment of beads with template DNA fragments using the Ion Chef^TM^ System (ThermoFisher Scientific). DNA-bound beads were loaded into the Ion 510 or 520 chip and subsequently sequenced using the Ion GeneStudio^TM^ S5 System (ThermoFisher Scientific).

### Sex concordance and reciprocal aneuploidy analysis

Sequencing run results were aligned to the sequence of the mouse genome (GRCm39) using the Burrows-Wheeler Aligner (BWA) package (https://bio-bwa.sourceforge.net/). Sequencing reads with a read count of >1000 were considered overrepresented and discarded from the analysis. Sequences with uniquely mapped reads (MAPQ score ≥20) were kept. A euploid male mouse (40, XY) was used to prepare a normal panel to reference other samples. Total read counts were normalized to 1 million counts per sample (excluding sex chromosomes) and were compared against the generated normal panel for every 10 Mb window previously analyzed to predict the karyotype of the sample. The fold change was calculated as a ratio that measures the degree of quantity change between the end result and the initial result (Creative Proteomics). Samples failed quality control if they had a proportion of reads mapped to the mouse genome that was <90% compared to the human genome and were excluded from downstream analysis (n = 1).

The observed copy number for each chromosome was obtained by multiplying the fold change by two (for autosomes) or one (for sex chromosomes). A designated copy number was determined based on both the observed copy number value and the interpretation of reciprocal aneuploidies in the egg/polar body pairs for the same chromosome. Copy number plots for the sequenced samples were then generated to analyze chromosome counts. As part of platform validation, sex concordance analysis was conducted by counting the number of X and Y chromosome copies present within a blastomere and comparing it with other blastomeres from the same embryo for platform validation. The same approach was also conducted between matched eggs and polar bodies (Fig. 1A). Reciprocal aneuploidy analysis was conducted by comparing chromatid copy numbers between matched eggs and polar bodies to determine whether additional chromosomes are present in one and reciprocally absent in the other. In normal MI, one of two homologous chromosomes (each consisting of two sister chromatids) is extruded from the oocyte to the polar body to generate two haploid gametes. Aneuploidies were categorized as either PSSC or NDJ. PSSC is defined as the presence of three chromatids in the egg and one chromatid in the polar body, or vice versa. NDJ is defined as all four chromatids remaining in the egg and no chromatids in the polar body, or vice versa. All karyotypes were curated based on nomenclature previously described for shallow NGS (McGowen-Jordan *et al.*, 2016). Individual matched egg and polar body pairs were categorized based on ploidy status: euploid, 1–3 chromosomal segregation errors, or ≥4 chromosomal segregation errors.

### Statistical analysis

Data are presented as the mean ± SEM, and each experiment was repeated two times. All results were graphed using Graphpad Prism Software Version 8.0.1 (La Jolla, CA, USA). The normal distribution of data was evaluated with the Shapiro–Wilk test. Analysis between groups of continuous variables was performed with Student’s *t*-test. Multiple comparisons were analyzed with a one-way ANOVA test or Kruskal–Wallis test followed by Tukey’s or Dunn’s multiple-comparison tests. Categorical variables were analyzed with Fisher’s exact test or chi-square test. *P*-values <0.05 were considered statistically significant. All total numbers of matched egg/polar body pairs used are specified in the figure legends.

## Results

### Blastomere sex concordance and polar body–egg reciprocal aneuploidies demonstrate accuracy of the NGS aneuploidy screening approach in mice

To validate a commercially available NGS platform for aneuploidy screening in the mouse model, autosome and sex concordance analyses were performed using mouse blastomeres (n = 21) from 4-cell embryos and matched egg and polar body pairs (Fig. 1A). As anticipated, NGS was able to detect euploid status by the presence of two copies of all autosomal chromosomes (1–19) and sex chromosomes (X and Y) in blastomeres as well as the egg and polar body (Supplementary Fig. S3). The presence of two X chromosomes or one X and one Y chromosome was detected in female and male blastomeres, respectively (Supplementary Fig. S3A and B). As expected, all tested blastomeres within the same embryo were 100% concordant in their sex chromosomes when compared to each other (Supplementary Fig. S3A and  B). Similarly, all matched eggs and polar bodies displayed the presence of two X chromosomes (Supplementary Fig. S3C). Together these findings validate the use of this NGS methodology for assessment of chromosome-specific ploidy status.

### Nocodazole treatment impairs meiotic progression in a dose-dependent manner

**Figure 2.**
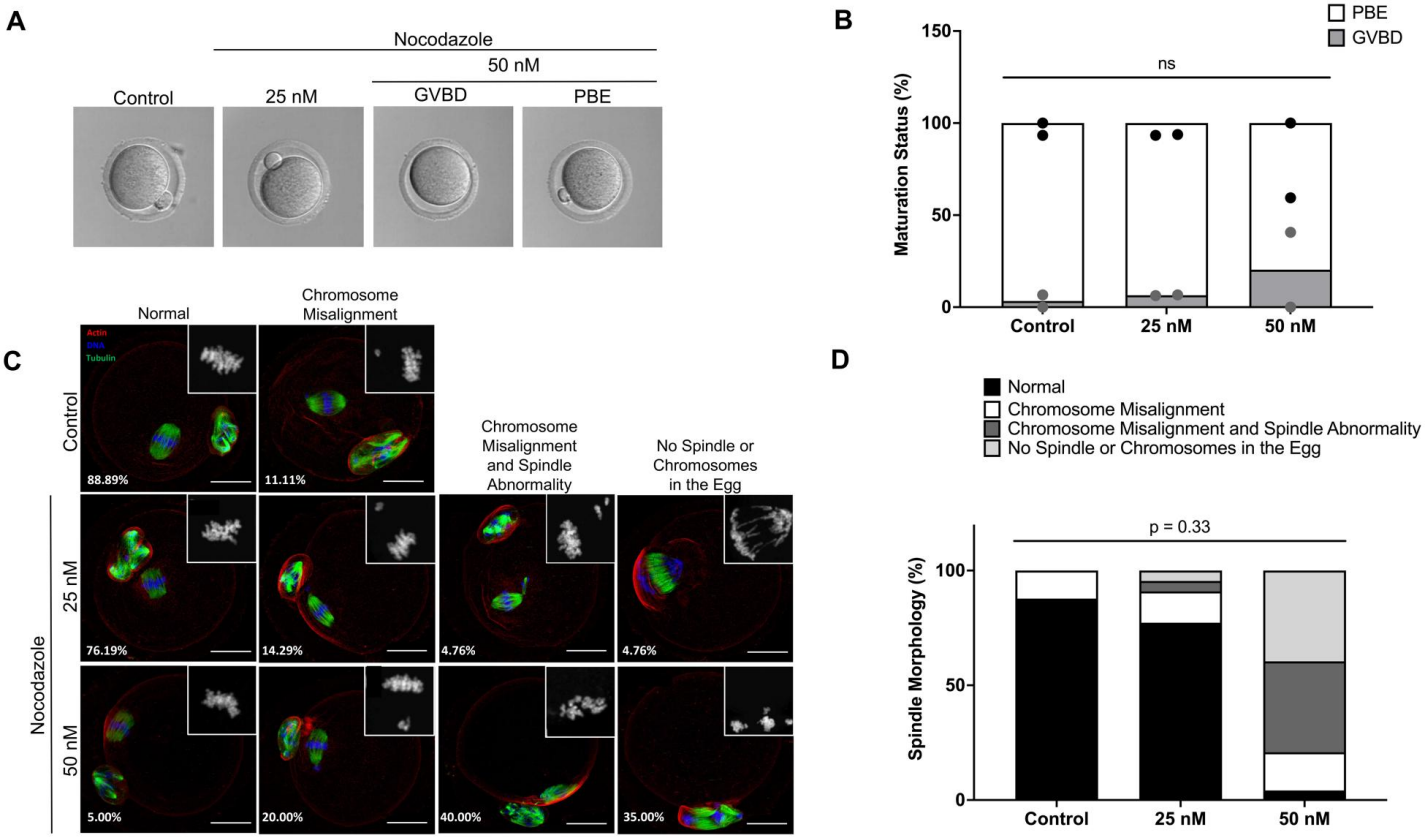
**The effect of Nocodazole on oocyte maturation and spindle morphology**. (**A**) Representative EmbryoScope+™ images of denuded oocytes treated with 25 or 50 nM Nocodazole following IVM. (**B**) Graph of meiotic progression for denuded oocytes treated with DMSO (control) and Nocodazole following IVM. Grey and black dots denote the percentage of oocytes at the GVBD or PBE stage, respectively, per replicate. (**C**) Representative immunofluorescent images showcasing the morphologies of control or Nocodazole-treated denuded oocytes during IVM. Actin (red), α-tubulin (green), and DNA (blue) were detected by immunocytochemistry. Scale bar = 25 µm. (**D**) Quantification of varying degrees of spindle abnormalities observed across control and Nocodazole-treated oocytes. N = 11–23 oocytes per treatment group across two replicates. GVBD, germinal vesicle breakdown; PBE, polar body extrusion; ns, not significant.

To investigate the relationship between aneuploidy and the morphokinetics of meiotic progression, we used Nocodazole, a known microtubule disruptor, to induce spindle abnormalities and chromosome segregation errors (Sun *et al.*, 2005; Yun *et al.*, 2014; Suebthawinkul *et al.*, 2022). Nocodazole treatment prevented a subset of oocytes from extruding a PB and reaching MII, especially in the 50 nM treatment group (Fig. 2A and B). As expected, given its role as a microtubule depolymerizing drug, Nocodazole treatment also resulted in phenotypes in the resulting MII eggs that ranged in severity, including chromosome misalignment, chromosome misalignment combined with spindle defects, and complete absence of chromosomes or spindles within the egg (Fig. 2C). Whereas most control eggs (88.89%) had normal bipolar MII spindles with chromosomes aligned along the metaphase plate, the prevalence of this morphology was reduced to 76.19% and 5.0% in the 25 and 50 nM Nocodazole groups, respectively (Fig. 2C and D). The proportion of severe phenotypes increased with Nocodazole treatment in a dose-dependent manner, although this trend was not significant (Fig. 2D). Oocytes treated with 50 nM Nocodazole exhibited a greater proportion of chromosome misalignment (20.0% vs 14.29% for 25 nM Nocodazole), chromosome misalignment and spindle abnormalities (40.0%, vs 4.76% for 25 nM Nocodazole), and complete extrusion of spindles and chromosomes into the polar body (35.0% vs 4.76% for 25 nM Nocodazole).

### Nocodazole treatment is associated with increased aneuploidy and disrupts the morphokinetic variables of meiotic maturation in a dose-dependent manner

**Figure 3.**
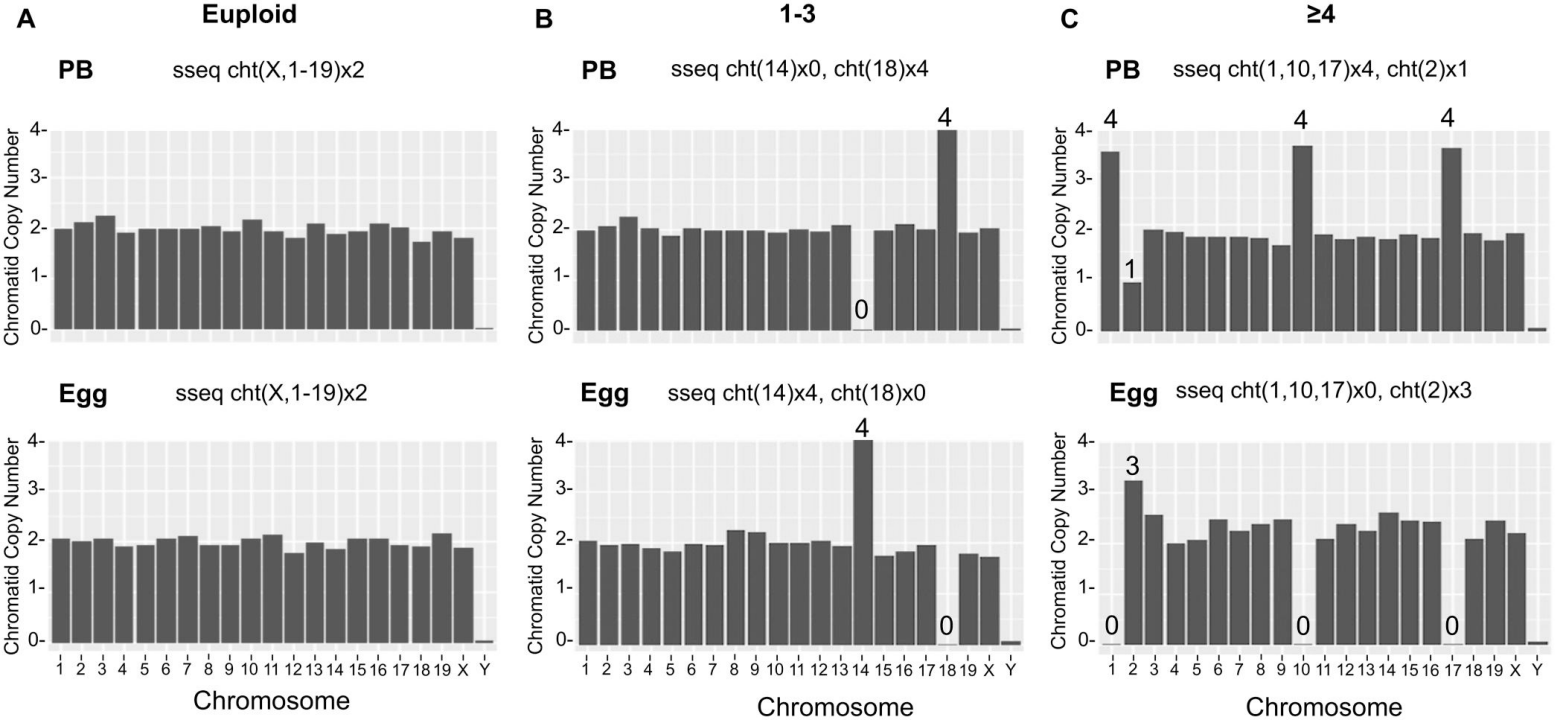
**Reciprocal aneuploidy analysis on matched MII oocyte and polar body pairs reveals nondisjunction (NDJ) of homologs and premature separation of sister chromatids (PSSC)**. Representative chromosome plots classifying the severity of reciprocal aneuploidy as (**A**) euploid, (**B**) 1–3 aneuploidy events, and (**C**) ≥4 reciprocal aneuploidy events. Chromosomes with abnormal copy numbers are labeled at the top of the bars. PB, polar body.

Given the observation that Nocodazole caused dose-dependent defects in spindle formation and chromosome alignment, we expected these phenotypes to translate into aneuploidy. Thus, to determine whether there was a relationship between morphokinetic variables of meiotic progression and aneuploidy, we performed IVM of oocytes in the EmbryoScope+™ in the presence or absence of two concentrations of Nocodazole and then analyzed the ploidy status of the resulting eggs and correlated this with morphokinetic variables of meiotic progression (Fig. 1B). Using the NGS platform on matched egg and polar body pairs, we were able to successfully determine the presence and type of reciprocal aneuploidy (Fig. 3 and Table 1). We categorized the gametes as: euploid, 1–3 chromosome segregation errors, or ≥4 chromosomal segregation errors (Fig. 3). We were able to detect specific types of aneuploidy including NDJ and PSSC (Fig. 3B and C and Table 1).

**Table 1.** Summary of karyotypes from aneuploid matched oocyte and polar body pairs.

Sample ID	Treatment group	Oocyte karyotype	Polar body karyotype	Aneuploidy type
Sample 1	25 nM	sseq cht(1)x4	sseq cht(1)x0	NDJ
Sample 2	50 nM	sseq cht(4)x4	sseq cht(4)x0	NDJ
Sample 3	50 nM	sseq cht(4)x4	sseq cht(4)x0	NDJ
Sample 4	50 nM	sseq cht(7)x0	sseq cht(7)x4	NDJ
Sample 5	50 nM	sseq cht(1)x1	sseq cht(1)x3	PSSC
Sample 6	50 nM	sseq cht(8,11)x0	sseq cht(8,11)x4	NDJ
Sample 7	50 nM	sseq cht(2,16,18)x4	sseq cht(2,16,18)x0	NDJ
Sample 8	50 nM	sseq cht(14)x4, cht(18)x0	sseq cht(14)x0, cht(18)x4	NDJ
Sample 9	50 nM	sseq cht(9)x0, cht(12,14)x4	sseq cht(9)x4, cht(12,14)x0	NDJ
Sample 10	50 nM	sseq cht(4,9,16,19)x0	sseq cht(4,9,16,19)x4	NDJ
Sample 11	50 nM	sseq cht(1,8)x0, cht(4,18)x4	sseq cht(1,8)x4, cht(4,18)x0	NDJ
Sample 12	50 nM	sseq cht(5)x4, cht(6,12,14)x0	sseq cht(5)x0, cht(6,12,14)x4	NDJ
Sample 13	50 nM	sseq cht(1,10,17)x0, cht(2)x3	sseq cht(1,10,17)x4, cht(2)x1	NDJ+PSSC
Sample 14	50 nM	sseq cht(X, 1-6,8-19)x0, cht(7)x4	sseq cht(X , 1-6,8-19)x4, cht(7)x0	NDJ
Sample 15	50 nM	sseq cht(X, 1-6,8-19)x0, cht(7)x4	sseq cht(X , 1-6,8-19)x4, cht(7)x0	NDJ
Sample 16	50 nM	sseq cht(X, 1-10,12,13,15-19)x0, cht(11,14)x3	sseq cht(X , 1-10,12,13,15-19)x4, cht(11,14)x1	NDJ+PSSC
Sample 17	50 nM	sseq cht(X, 1-4, 6-17,19)x0, cht(5,18)x3	sseq cht(X , 1-4, 6-17,19)x4, cht(5,18)x1	NDJ+PSSC
Sample 18	50 nM	sseq cht(1,3-8,10-13,15-19)x0, cht(X, 2,9,14)x3	sseq cht(1,3-8,10-13,15-19)x4, cht(X , 2,9,14)x1	NDJ+PSSC
Sample 19	50 nM	sseq cht(X, 1,3-9,12,13)x3, cht(2,10,11,14,16-19)x0, cht(15)x1	sseq cht(X , 1,3-9, 12,13)x1, cht(2,10,11,14,16-19)x4, cht(15)x3	NDJ+PSSC
Sample 20	50 nM	sseq cht(X, 1-4,6-8,11-17,19)x0, cht(5)x4, cht(9,10,18)x3	sseq cht(X , 1-4,6-8,11-17,19)x4, cht(5)x0, cht(9,10,18)x1	NDJ+PSSC

NDJ, non-disjunction; PSSC, premature separation of sister chromatids.

**Figure 4.**
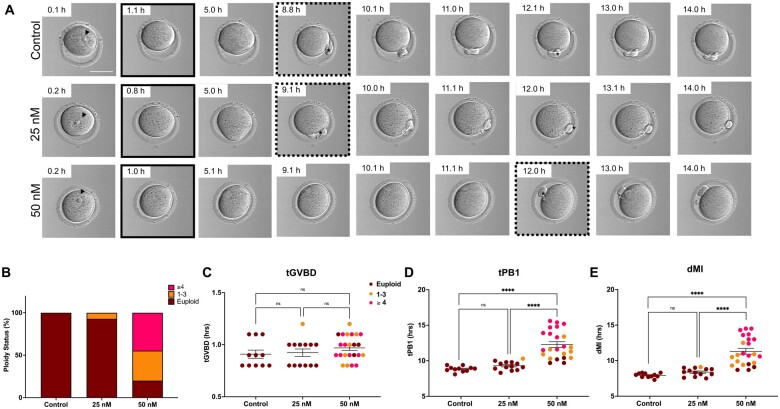
**The effect of Nocodazole on ploidy status and morphokinetic variables of meiotic progression**. (**A**) Representative EmbryoScope+™ images of the control and Nocodazole-treated oocytes during IVM. Germinal vesicle and timepoint of GVBD are indicated by arrows (black) and solid black squares, respectively. Timepoint of the first extruded polar body (asterisk) is indicated by the dotted squares. (**B**) Graph of the proportion of oocytes within each reciprocal aneuploidy categorization (euploid, 1–3, or ≥4 aneuploidy events). (**C–E**) Quantification of (C) tGVBD, (D) tPB1, and (E) dMI in oocytes treated with DMSO (control) or different concentrations of Nocodazole. N = 11 (control), 13 (25 nM), and 23 (50 nM) oocytes across two replicates. GVBD, germinal vesicle breakdown; tGVBD, time to GVBD; tPB1, time to extrusion of the first polar body; dMI, duration of meiosis I; ns, not significant. ****P < 0.0001.

Treatment with Nocodazole induced aneuploidy at both 25 and 50 nM, with a greater incidence observed at the higher concentration (Fig. 4B and Table 1). Although the proportion of euploid eggs was similar between the control and the 25 nM group (100% and 92.30%, respectively), only 17.40% of the eggs were euploid in the 50 nM group (Fig. 4B). Of the aneuploid eggs treated with Nocodazole, 13/20 exhibited only NDJ, 1/20 exhibited only PSSC, and 6/20 exhibited both NDJ and PSSC (Table 1). The severity of the aneuploidy increased with the concentration of Nocodazole, with mis-segregation of more than four chromosomes only being observed at the highest dose (Fig. 4B and Table 1). Using the NGS platform, we were able to detect chromosome-specific mis-segregation events and their prevalence across both types of meiotic errors. NDJ events were observed among all chromosomes (Supplementary Fig. S4A), whereas PSSC was observed among all chromosomes except 16, 17, and 19 (Supplementary Fig. S4B).

Nocodazole treatment did not affect the tGVBD (0.91 ± 0.04, 0.92 ± 0.04, 0.97 ± 0.02 h for the control, 25 nM, and 50 nM of Nocodazole, respectively, *P* = 0.342) (Fig. 4A and C). In contrast, tPB1 was increased in the 50 nM group relative to the control and the 25 nM group (12.29 ± 0.41 vs 8.84 ± 0.11 and 9.31 ± 0.17 h, respectively, *P* < 0.0001) (Fig. 4D). Consequently, the prolonged tPB1 in the 50 nM group resulted in a significantly longer dMI in this group relative to the other experimental cohorts (11.32 ± 0.41 vs 7.94 ± 0.09 and 8.38 ± 0.15 h for the control and 25 nM Nocodazole, respectively, *P* < 0.0001). These results demonstrate that pharmacological inhibition of spindle formation by Nocodazole can perturb the morphokinetics of meiotic progression, and particularly the timing of polar body extrusion, in a dose-dependent manner and are consistent with our previous findings (Suebthawinkul *et al.*, 2022).

**Figure 5.**
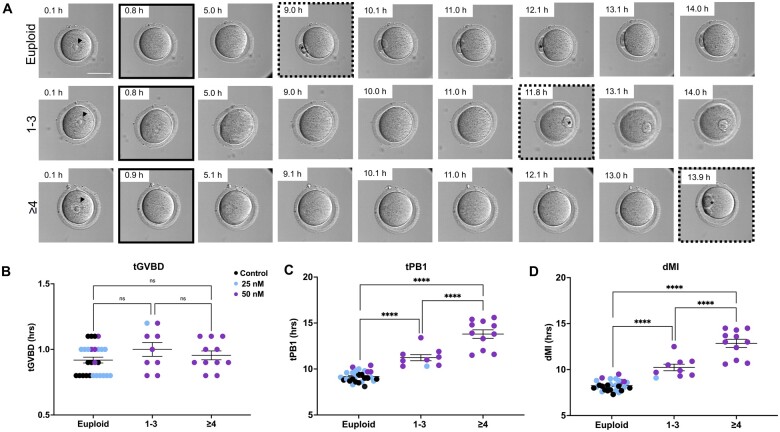
**The effect of ploidy status on morphokinetic variables of meiotic progression**. (**A**) Representative EmbryoScope+™ images of oocytes with different ploidy status during IVM. Germinal vesicle and timepoint of GVBD are indicated by arrows (black) and solid black squares, respectively. Timepoint of the first extruded polar body (asterisk) is indicated by the dotted squares. (**C–E**) Quantification of (C) tGVBD, (D) tPB1, and (E) dMI in oocytes of different ploidy status. N = 27 (euploid), 9 (1–3 aneuploidy events), and 11 (≥4 aneuploidy events) oocytes across two replicates. GVBD, germinal vesicle breakdown; tGVBD, time to GVBD; tPB1, time to extrusion of the first polar body; dMI, duration of meiosis I; ns, not significant. ****P < 0.0001.

We then compared morphokinetic variables of meiotic maturation based on ploidy status (Fig. 5). The presence of reciprocal aneuploidy did not affect the tGVBD (0.92 ± 0.02, 1.00 ± 0.05, 0.95 ± 0.03 h for euploid, 1–3, and ≥4 aneuploidy events, respectively, *P* = 0.225) (Fig. 5A and B). However, tPB1 was delayed in eggs with reciprocal aneuploidy (9.19 ± 0.11, 11.23 ± 0.33, and 13.80 ± 0.46 h for euploid, 1–3, and ≥4 chromosome segregation errors, respectively, *P* < 0.0001) (Fig. 5C). This prolonged tPB1 also translated to a longer dMI in aneuploid eggs compared to euploid eggs (8.27 ± 0.11, 10.23 ± 0.36, and 12.85 ± 0.45 h for euploid, 1–3, and ≥4 chromosome segregation errors, *P* < 0.0001). In addition, the prolonged tPB1 and dMI were also significantly longer in eggs with ≥4 chromosome segregation errors compared to those with only 1–3 (*P* < 0.001), suggesting that these readouts are sensitive enough to differentiate based on severity of aneuploidy. Although the sample size was limited, eggs with only a single aneuploidy event exhibited a longer tPB1 and dMI compared to euploid eggs (Supplementary Fig. S5A–C). Overall, these findings demonstrated that chromosome segregation errors are paralleled by significantly altered morphokinetic variables that can be tracked non-invasively.

## Discussion

In this study, we used a comprehensive chromosome screening method to investigate the relationship between morphokinetic variables of meiotic progression and aneuploidy using mouse oocytes. The ability to reverse translate a clinical workflow that is typically used in preimplantation genetic testing for aneuploidy (PGT-A) is critical for basic science studies in the mouse model system, as it allows the assessment of chromosome segregation errors at the individual chromosome level and a resolution that is high enough to clearly distinguish errors due to NDJ or PSSC. This method has now been applied to mouse eggs and embryos and represents an important advance over conventional approaches (Treff *et al.*, 2016; Tao *et al.*, 2017). For example, it is often difficult to accurately identify specific chromosomes using chromosome spreading and fluorescence *in situ* hybridization (FISH). Traditional chromosome spreading requires cell dissolution and is thus prone to chromosome loss (Hoo and Bowles, 1971; Duncan *et al.*, 2009). The FISH technique is used extensively to investigate aneuploidy in mouse models but can only detect a few chromosomes simultaneously (Zudova *et al.*, 2003; Lechniak *et al.*, 2007; Hornak *et al.*, 2012). Even *in situ* chromosome spreading, a technique which allows more accurate chromosome counts, is subject to human errors and fails to identify chromosome-specific aneuploidy (Duncan *et al.*, 2009; Treff *et al.*, 2016).

We used Nocodazole at different concentrations to induce a spectrum of aneuploidy based on its known role in regulating microtubules and spindle dynamics (Jordan *et al.*, 1992). Oocytes treated with the higher concentration of Nocodazole (50 nM) during IVM exhibited abnormal morphokinetics in meiotic maturation, specifically a longer time to polar body extrusion (PBE) and duration of MI. The eggs in this group also had the highest degree of aneuploidy. Of note, in a previous study using a mouse model of physiologic reproductive aging, we did not observe differences in the morphokinetics of meiotic progression between euploid and aneuploid eggs when ploidy status was assessed using an *in situ* chromosome spreading method (Suebthawinkul *et al.*, 2023). The discrepancy in these results could be because the aneuploidy in the physiologic aging mouse model was not as severe as that achieved by Nocodazole. The incidence of aneuploidy was reported to be 24–29% in reproductively old mice, but in the present model, the aneuploidy incidence was as high as 82.6% for the 50 nM group and included complex aneuploidies involving multiple chromosomes (Suebthawinkul *et al.*, 2023). It is also possible that the underlying mechanisms leading to chromosome segregation errors differ, which explains the prevalence of NDJ over PSSC in our study. PSSC has been reported to be more prevalent than NDJ in the oocytes with increased reproductive age (Gruhn *et al.*, 2019; Mikwar *et al.*, 2020). This is primarily due to the loss of chromosome cohesion between the centromeres of sister chromatids and the chromosome arms during meiotic division (Nagaoka *et al.*, 2012; Mikwar *et al.*, 2020). In contrast, Nocodazole disrupts the microtubule cytoskeleton and spindle formation, resulting in mis-segregation or loss of homologous chromosome pairs and, therefore, a preference for NDJ (Decordier *et al.*, 2008; Duncan *et al.*, 2009). In addition, acrocentric chromosomes have been shown to be more prone to NDJ (Gruhn *et al.*, 2019). Given that mice only have acrocentric chromosomes, our findings are consistent with the expected increased prevalence of NDJ versus PSSC. Although the primary mode-of-action of Nocodazole in mouse oocytes is microtubule depolymerization, we cannot rule out potential effects on other biological processes.

Using the NGS platform, we were able to evaluate the types of meiotic aneuploidy events at a chromosome-specific resolution. NDJ events occurred across all chromosomes whereas PSSC events were present in most but not all chromosomes. Notably, the lack of PSSC events on certain chromosomes, such as chromosomes 16, 17, and 19, support previous observations in aneuploid eggs from reproductively aged mice (Treff *et al.*, 2016). Despite the differences in the origin of aneuploidy, these observations suggest that certain chromosomes may be more resilient against segregation errors leading to PSSC. However, future studies leveraging the NGS platform on a larger number of eggs are required to more comprehensively compare aneuploidy generated by physiological aging versus pharmacological treatment.

The aneuploid eggs in our study exhibited a significantly extended time to PBE and duration of MI relative to euploid eggs, and this may be due to activation of the spindle assembly checkpoint (SAC). The SAC regulates meiosis by monitoring the proper attachment and segregation of chromosomes on the spindle. In the presence of unstable or incorrect kinetochore–microtubule attachments, SAC proteins inhibit the activation of anaphase-promoting complex (APC), to delay MI exit and allow more time to repair the erroneous attachment (Collin *et al.*, 2013; Lane and Jones, 2014; Lane and Kauppi, 2019). However, instead of eliciting an ‘all or nothing’ response, the SAC may only provide a gentle braking force on the APC (Collin *et al.*, 2013; Lane and Kauppi, 2019). This limited checkpoint activity may explain why the female gametes are more prone to develop aneuploidy and generate mis-segregated chromosomes despite the extended duration of MI. Our model system can serve as a platform for identifying compounds that disrupt meiotic maturation and induce aneuploidy. Whether extended time to PBE is a common feature of all compounds that induce aneuploidy is an important area for further investigation.

The use of time-lapse technology to study the association between morphokinetics and ploidy status has been mostly in human embryos. Using time-lapse technology, aneuploid embryos have been found to exhibit delayed timing in embryonic development, including initiation of compaction and initiation of blastocyst formation, expansion, and hatching (Campbell *et al.*, 2013; Minasi *et al.*, 2016). Differences in the patterns of ooplasmic movements before the first cytokinesis event have also been distinguished between euploid and aneuploid embryos (Serrano-Novillo *et al.*, 2023). These results suggest that aneuploid embryos are delayed in various key developmental processes, which is consistent with our observations of delayed meiotic progression in mouse oocytes. In addition, our findings suggest that morphokinetic variables of oocyte maturation are, in principle, sensitive enough to detect single chromosome aneuploidies which may have clinical relevance (i.e. trisomies). However, future research focused on larger samples of eggs with a single aneuploidy is required to comprehensively evaluate the detection limit of this platform.

The ability to detect relationships between morphokinetic variables of meiotic progression and ploidy status may inform additional opportunities. For example, the morphokinetic variables of meiotic maturation can be used as a screening method for drug discovery, since compounds that inhibit or significantly delay meiotic maturation could have utility as non-hormonal female contraceptives. However, aneuploidy would have to be carefully evaluated in a safety assessment. Being able to accurately assess aneuploidy also improves the utility of the mouse as a model system. For example, the mouse embryo assay has been a valuable tool for analyzing the effectiveness and potential toxicity of culture media, instruments, cryopreservation, etc (Esfandiari and Gubista, 2020). Currently, these consumables and instruments are analyzed by the morphological qualities of the embryo. Utilizing an NGS platform in these tests could provide advantageous chromosome-specific information for how certain products may affect embryo ploidy status, ultimately informing optimal conditions for ART.

Morphokinetic variables of meiotic progression and ploidy status may also be relevant in the clinical context of human IVM, which is a method that has promise for patients at risk of ovarian hyperstimulation syndrome (Gremeau *et al.*, 2012; Walls *et al.*, 2015) and those undergoing ovarian tissue cryopreservation for fertility preservation (Fasano *et al.*, 2017; Shirasawa and Terada, 2017). In addition, immature oocytes typically collected as part of standard IVF protocols can be *in vitro* matured to increase the yield of mature eggs. A better understanding of the morphokinetics of human IVM is warranted to harness this technology to improve outcomes. However, human oocytes must be *in vitro* matured with cumulus cells intact which will likely obscure the ability to track the morphokinetic variables of meiotic maturation specifically within the oocyte. There is also likely critical information to be gleaned by tracking the morphokinetics of cumulus expansion as we have shown in the mouse (Suebthawinkul *et al.*, 2022, 2023). Monitoring the morphokinetics of intact human COCs during IVM is an exciting opportunity but will require engineering new time-lapse imaging modalities with a large enough field of view.

Taken together, we combined the use of time-lapse imaging with a comprehensive chromosome screening method to investigate the relationship between aneuploidy and morphokinetic dynamics. We used Nocodazole to induce aneuploidy and found that eggs with chromosomal segregation errors had a prolonged time to PBE and duration of MI during meiotic maturation. Whether this holds true in human IVM requires further investigation; however, our results provide significant insight into the non-invasive assessment of oocyte quality that could be applied in clinical and pharmaceutical settings. Additionally, given the challenges of obtaining human oocytes for research, the mouse serves as an important model system for the study of meiotic maturation and aneuploidy.

## Supplementary Material

hoae023_Supplementary_Data

## Data Availability

All original data in this publication are available upon reasonable request to the corresponding author.
