# Supplementary material for: The severity of meiotic aneuploidy is associated with altered morphokinetic variables of mouse oocyte maturation
Source: Hum Reprod Open. 2024 Apr 23;2024(2):hoae023. doi: 10.1093/hropen/hoae023 (PMC11099657; doi:10.1093/hropen/hoae023)
Supplement: hoae023_Supplementary_Data [file hoae023_supplementary_data.docx]

**The severity of meiotic aneuploidy is associated with altered morphokinetic variables of mouse oocyte maturation**

Yiru Zhu, Catherine R. Kratka, Jeffrey Pea, Hoi Chang Lee, Caroline E. Kratka, Jia Xu, Diego Marin, Nathan R. Treff, Francesca E. Duncan

**Supplementary Figures**

Supplementary Figure S1. Image annotation of germinal vesicle breakdown (GVBD) in oocytes undergoing in vitro maturation (IVM) on EmbryoScope+^TM^.

Supplementary Figure S2. Image annotation of polar body extrusion (PBE) in oocytes undergoing in vitro maturation (IVM) on EmbryoScope+^TM^.

Supplementary Figure S3. Validation of next-generation sequencing (NGS) chromosome analysis using mouse blastomeres and matched meiosis II (MII) oocyte and polar body pairs.

Supplementary Figure S4. Frequencies of chromosome-specific aneuploidy events in Nocodazole-treated oocytes.

Supplementary Figure S5. The effect of single aneuploidy event on morphokinetic variables of meiotic progression.

**Supplementary Figure S1. Image annotation of germinal vesicle breakdown (GVBD) in oocytes undergoing in vitro maturation (IVM) on EmbryoScope+^TM^. (A)** Representative EmbryoScope+^TM^ images of control denuded oocytes undergoing GVBD during IVM. Images are taken approximately 10-20 minutes per oocyte with the shown timepoints representing the timepoint prior (0.9 h) and after (1.1 h) GVBD. The focal plane at a specific timepoint selected for assessment of morphokinetic variables is indicated by the solid black squares. (**B)** Zoomed in representative EmbryoScope+^TM^ images of control denuded oocytes with intact GV (0.9 h) and after GVBD (1.1 h).

**Supplementary Figure S2. Image annotation of polar body extrusion (PBE) in oocytes undergoing in vitro maturation (IVM) on EmbryoScope+^TM^. (A)** Representative EmbryoScope+^TM^ images of control denuded oocytes undergoing extrusion of the first polar body (PBE) during IVM. Images are taken approximately 10-20 minutes per oocyte with the shown timepoints representing the timepoints before (8.7 h) and during (8.9 h) polar body appearance (PBA) as well as the timepoints before (9.0 h) and after (9.2 h) the complete PBE. The focal plane at a specific timepoint selected for assessment of morphokinetic variables is indicated by the solid black squares. (**B)** Zoomed in representative EmbryoScope+^TM^ images of control denuded oocytes before and after the complete PBE.


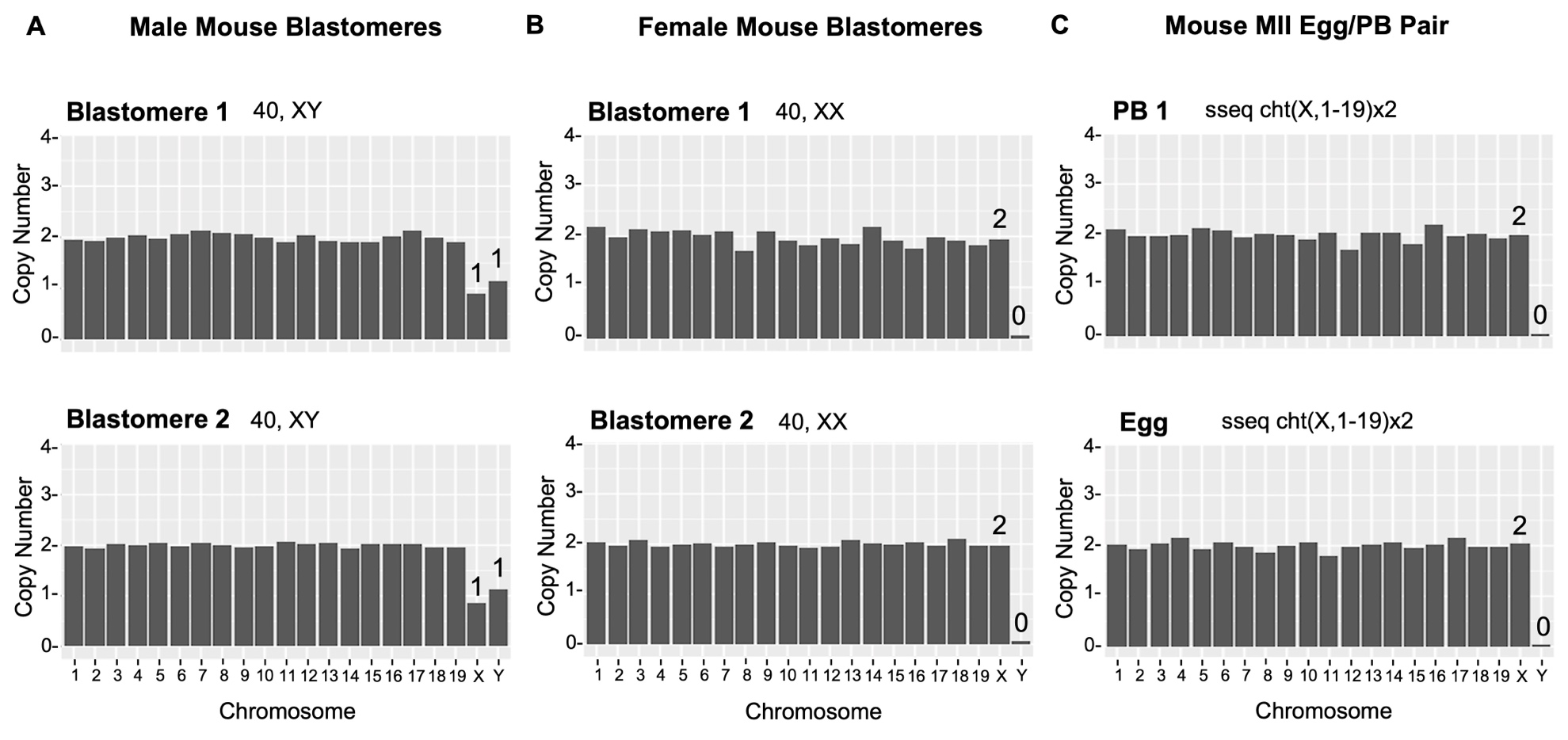


**Supplementary Figure S3. Validation of next-generation sequencing (NGS) chromosome analysis using mouse blastomeres and matched meiosis II (MII) oocyte and polar body pairs.** (**A-C**) Representative chromosome number plots for (A) male mouse blastomeres, (B) female mouse blastomeres, and (C) matched MII oocyte and polar body pairs. Comparison of blastomeres were conducted using those from the same embryo.

**Supplementary Figure S4. Frequencies of chromosome-specific aneuploidy events in Nocodazole-treated oocytes.** (**A-B**) Quantification of mis-segregation errors on each chromosome in aneuploid oocytes with either (A) non-disjunction (NDJ) or (B) premature separation of sister chromatids (PSSC).

**B**

**A**

**C**


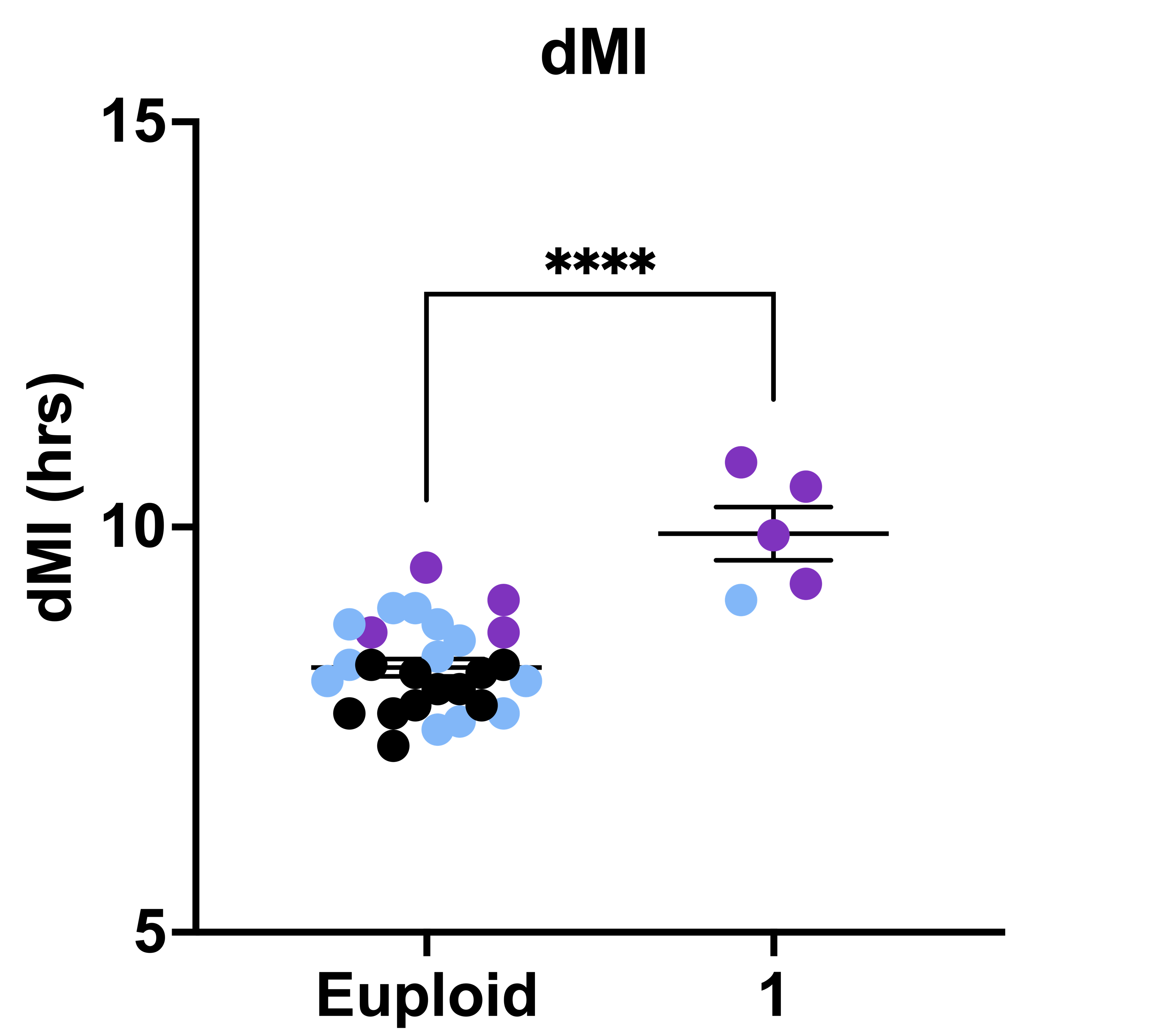

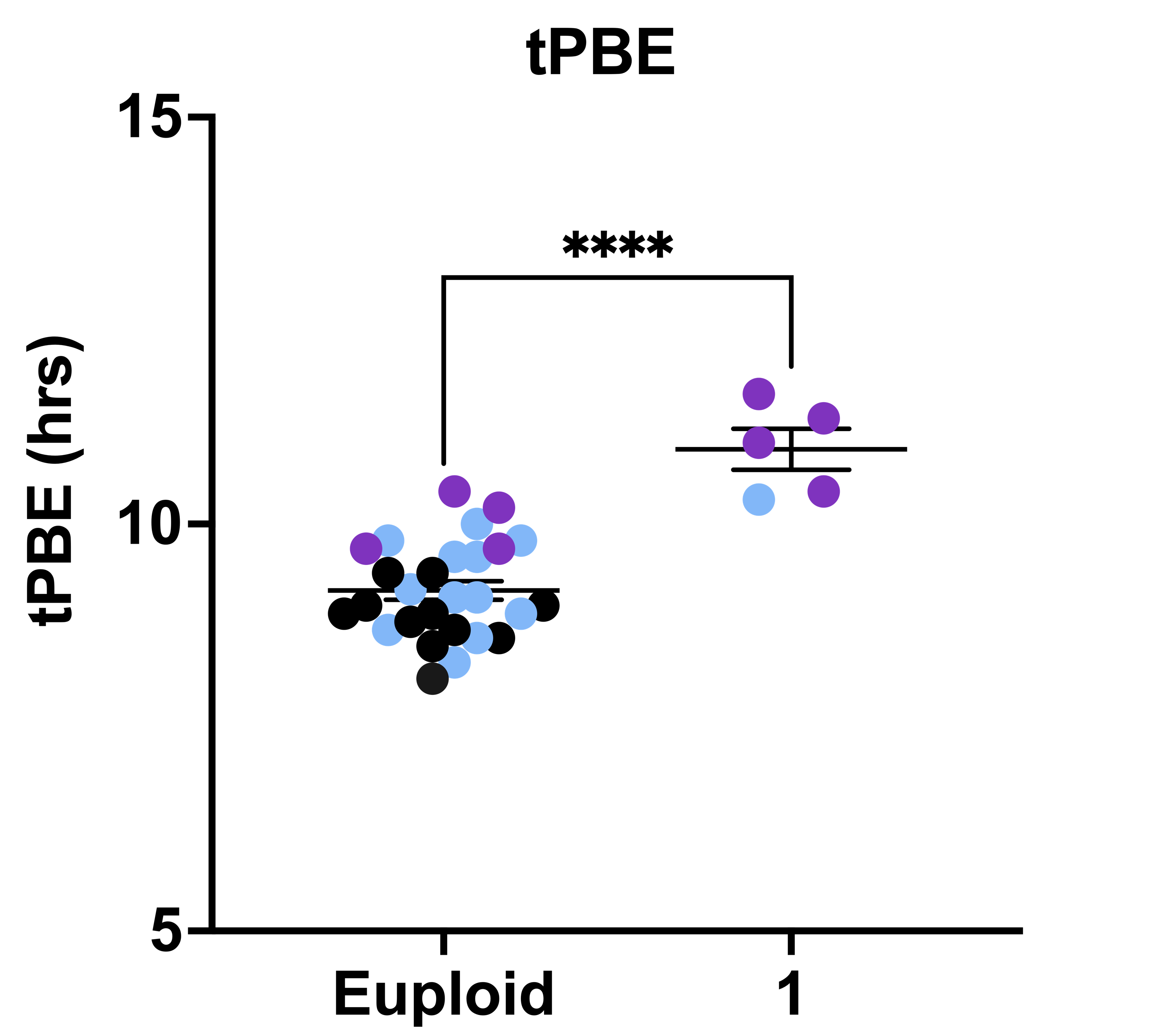

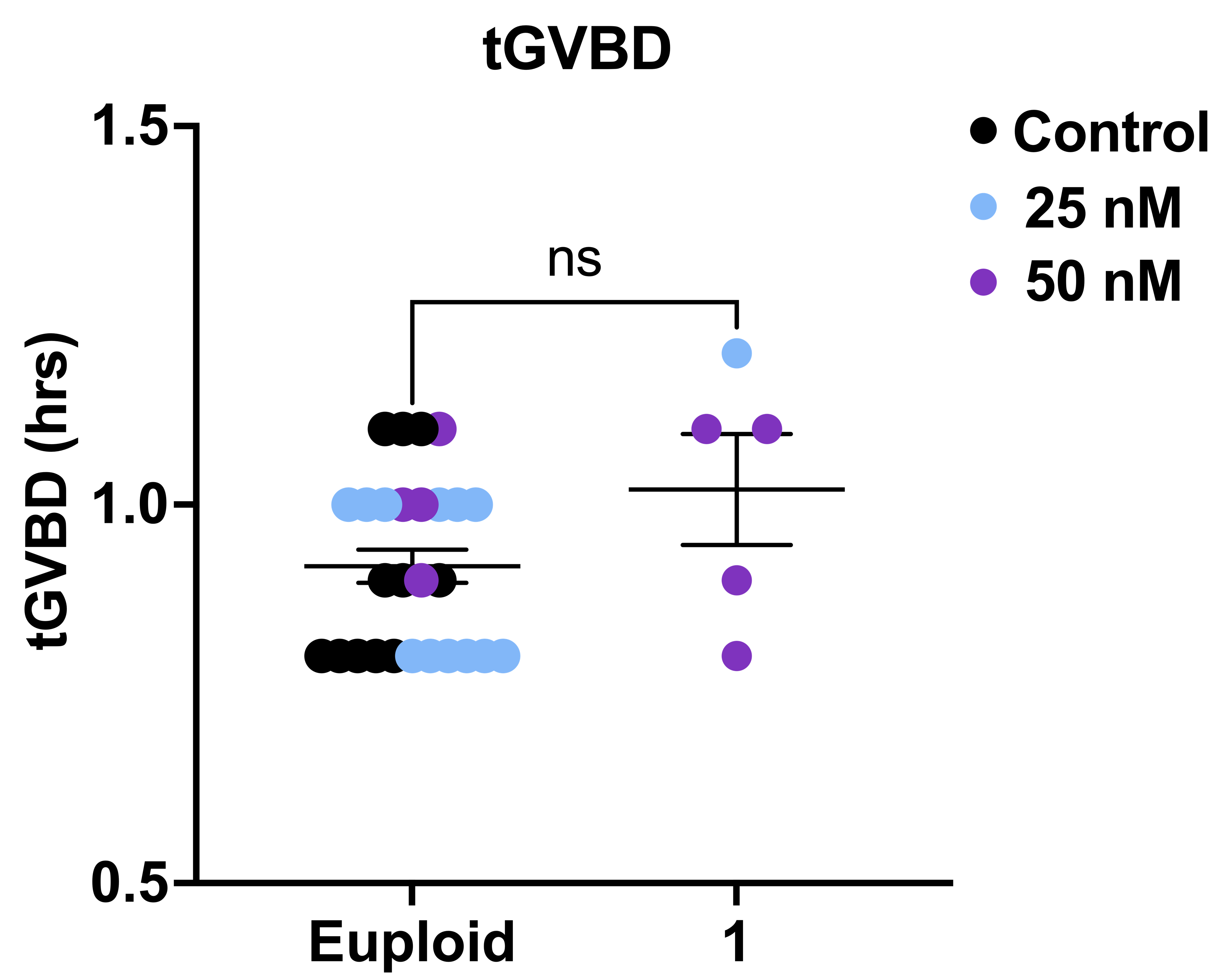


**tPB1**

**Supplementary Figure S5. The effect of single aneuploidy event on morphokinetic variables of meiotic progression.** (**A-C**) Quantification of (A) time to GVBD (tGVBD), (B) time to extrusion of the first polar body (tPB1), and (C) duration of meiosis I (dMI) between euploid oocytes (N=27) and oocytes with one aneuploidy event (N=5). ns = not significant. **** = p< 0.0001.
